# Healthcare workers’ infection risk perceptions of aerosol-generating procedures and affective response

**DOI:** 10.1017/ash.2022.276

**Published:** 2023-02-15

**Authors:** Lauren E. Benishek, Lewis J. Radonovich, Brie H. Blackley, David N. Weissman

**Affiliations:** 1 Armstrong Institute for Patient Safety and Quality, Johns Hopkins School of Medicine, Baltimore, Maryland; 2 Respiratory Health Division, National Institute for Occupational Safety and Health, Morgantown, West Virginia

## Abstract

**Objective:**

To understand healthcare worker (HCW) perceptions of infection risk associated with aerosol-generating procedures (AGPs) and their affective response to performing AGPs.

**Design:**

Systematic review.

**Methods:**

Systematic searches of PubMed, CINHAL Plus, and Scopus were conducted using combinations of selected keywords and synonyms. To reduce bias, titles and abstracts were screened for eligibility by 2 independent reviewers. Also, 2 independent reviewers extracted data from each eligible record. Discrepancies were discussed until consensus was reached.

**Results:**

In total, 16 reports from across the globe were included in this review. Findings suggest that AGPs are generally perceived to place HCWs at high risk of becoming infected with respiratory pathogens and that this perception stimulates a negative affective response and hesitancy to participate in the procedures.

**Conclusions:**

AGP risk perception are complex and context dependent but have important influences on HCW infection control practices, decision to participate in AGPs, emotional welfare, and workplace satisfaction. New and unfamiliar hazards paired with uncertainty lead to fear and anxiety about personal and others’ safety. These fears may create a psychological burden conducive to burnout. Empirical research is needed to thoroughly understand the interplay between HCW risk perceptions of distinct AGPs, their affective responses to conducting these procedures under various conditions, and their resulting decision to participate in these procedures. Results from such studies are essential for advancing clinical practice; they point to methods for mitigating provider distress and better recommendations for when and how to conduct AGPs.

The coronavirus disease 2019 (COVID-19) pandemic strained supply chains, disrupted healthcare operations, and tremendously affected the well-being of healthcare workers (HCWs).^1,2^ The pandemic necessitated unexpected and urgent adaptations to established workflows to reduce infection risk and slow the spread of severe acute respiratory coronavirus virus 2 (SARS-CoV-2), the virus causing COVID-19. Rapidly implemented policy and practice changes led to increased utilization of protective measures and equipment, such as N95 respirators, depleting or exhausting supplies of items that are typically readily available. Lack of evidence-based information regarding the severity, susceptibility, and transmission of COVID-19 led to uncertainty and confusion. HCW stress, anxiety, and depressive symptoms increased substantially.^3^ Emotional distress among HCWs^4^ was associated with fear of self-infection, fear of carrying the virus home to family and friends,^5^ decreases in patient care quality, and stigmatization.^6^ Fear was fueled by uncertainty regarding contagion risk and best practices for controlling an emerging virus.

Early in the pandemic, infection transmission routes were less well understood than at present; insufficient evidence existed to understand whether and to what extent SARS-CoV-2 could be transmitted from person to person by very small particles, often referred to as aerosols or droplet nuclei. In July 2020, the World Health Organization (WHO) issued a scientific brief that underscored aerosol-generating procedures (AGPs) as a source of transmission.^7^ Yet the amount of virus required for transmission, sometimes called the inoculum size, and many other factors that can influence human transmission were not sufficiently studied to draw conclusions.^8^ HCWs remained uncertain about the magnitude of risks associated with common patient care practices, including AGPs.

The Centers for Disease Control and Prevention has described AGPs as “… procedures performed on patients [that] are more likely to generate higher concentrations of infectious respiratory aerosols than coughing, sneezing, talking, or breathing.”^9^ However, AGP definitions and criteria are evolving. More recently, AGPs have been defined as “medical procedures that can result in the release of aerosols from the respiratory tract” that are “high risk of aerosol generation and increased risk of transmission.”^10^ Participation in AGPs has been documented to be a significant risk factor for transmission of highly contagious pathogens to HCWs.^11,12^ Yet, a large gap remains in our understanding of which and to what extent AGPs confer an increased risk of respiratory pathogen transmission via small-particle aerosols from patient to HCWs performing AGPs or that are present in spaces where they are performed.^13^ Insufficient evidence exists regarding which procedures or activities actually create small-particle aerosols of patient-derived material as opposed to simply being associated with increased risk for transmission via other mechanisms, such as via body fluid sprays or proximity to an infected patient. Emerging evidence suggests that proximity, duration, viral load, and room ventilation are primary risk factors for infection transmission, which may explain why procedures requiring close contact with patients increase transmission risk.^14^

Classical representations of behavior under uncertainty hold that beliefs—including perceptions—are key determinants of decisions related to money, health, life duration, and approval.^15^ Uncertainty especially affects decision making regarding health and welfare when most outcome probabilities are ambiguous and not objectively known.^16^ Without clear understanding of respiratory pathogen transmission risks associated with specific AGPs, HCWs face uncertainty about how to protect themselves and others. In many instances, they resort to presuming transmission risk based on personal experience, anecdotal evidence, and historical perceptions that AGPs pose higher exposure and infection risk to HCWs.

Although risk preferences and tolerance vary across individuals,^17^ attitudes mediate the relationship between perceptions and behavior.^18^ HCW risk perceptions will influence their cognitive (ie, beliefs) and affective (ie, feelings) attitudes regarding the appropriateness of conducting AGPs under various conditions. In turn, these attitudes shape their behaviors, influencing their willingness to conduct AGPs.^18^ Furthermore, because behavior reciprocally influences attitudes, HCW decisions to conduct AGPs or not can influence their emotional state. HCWs may experience moral distress when weighing personal safety against conducting potentially life-saving AGPs, such as emergent endotracheal intubation or cardiopulmonary resuscitation. Risk uncertainty may compound distress by leaving HCWs questioning whether prioritizing personal safety (and thereby protecting their ability to provide care to others) is ethical or justified. If risk perceptions are incorrect, HCWs may either compromise their own health unwittingly or unnecessarily modify care to levels that do not meet current standards or even delay or deny care to patients in need. The resulting conundrum may further distress HCWs and exacerbate symptoms of stress, anxiety, and depression.

Given the interplay of these phenomena, we sought to elucidate what is known about HCW perceptions of AGP risk and their affective response to those perceptions. To do so, we conducted a systematic review of the published literature about HCW reactions to conducting AGPs. Here, we present the results of our review.

## Methods

Between March 2 and April 10, 2021, we conducted systematic searches in PubMed, CINHAL Plus, and Scopus using combinations of the following key words and synonyms in conjunction with the controlled vocabulary of the database: “risk,” “risk perception,” “anxiety,” “stress,” “concern,” “fear,” “health personnel,” “aerosol generation,” “aerosol procedure,” and “aerosol generating procedure.” In total, 504 unique records were retrieved. We examined references in the included papers for additional studies. After those selections were added and duplicates were removed, 596 distinct records remained for screening.

**Fig. 1.**
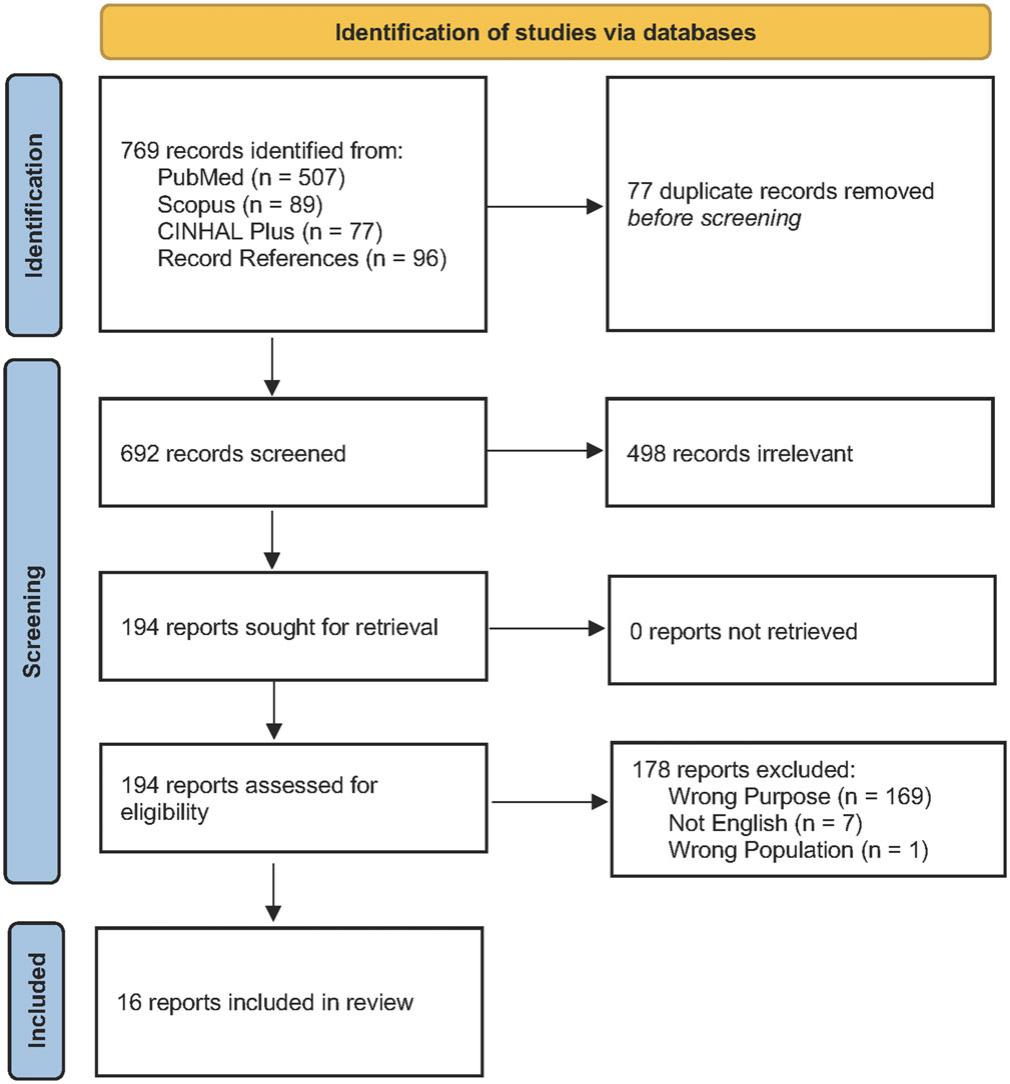
Preferred Reporting Items for Systematic Reviews and Meta-Analyses (PRISMA) Flowchart

To reduce the risk of bias, titles and abstracts were screened for eligibility by 2 independent reviewers (L.E.B. and D.W.). For the purposes of this paper, we defined HCW as any type of worker whose occupation is engaged directly or indirectly in providing healthcare services to patients. Papers describing perceived risk of adverse outcomes posed to HCWs while conducting an AGP or affective (eg, cognitions, attitudes, and emotions) responses of HCWs to perceived risk were included. Articles were excluded if they did not meet these inclusion criteria (eg, articles focused on non-HCWs, such as bystanders administering CPR, were not included) or were not written in English (Fig. 1). Articles were not excluded on the basis of publication type, presence of original data, or study design. All coauthors participated in data extraction as independent reviewers. Two independent reviewers extracted data from each record. Discrepancies were discussed by all coauthors until consensus was reached.

## Results

### Report characteristics

We included 16 reports in this review (Table 1).^19–34^ Publication date of the reports ranged from 1990–2022. Report settings included the United States (*n* = 7), Italy (*n* = 3), the United Kingdom (*n* = 2), Saudi Arabia (*n* = 2), Australia (*n* = 1), Cambodia (*n* = 1), Canada (*n* = 1), China (*n* = 1), India (*n* = 1), Indonesia (*n* = 1), Malaysia (*n* = 1), New Zealand (*n* = 1), Spain (*n* = 1), Taiwan (*n* = 1), Thailand (*n* = 1), Turkey (*n* = 1), and West Africa (*n* = 1). Included reports were either conceptual (*n* = 3) or empirical (*n* = 13). Conceptual reports were published as commentaries. Also, 12 empirical reports leveraged a cross-sectional survey research design; 1 study reported results from a semistructured interview study.

**Table 1.** Descriptions of Selected Peer-Reviewed Publications

Citation and Purpose	Report Type and Research Design	Disease	AGP of Interest	Population	Setting
**Ornato, Hallagan, McMahan, Peeples, & Rostafinski (1990)** ^19^					
Assessment of attitudes, beliefs, and behaviors with respect to the training and performance of MTM ventilation	Empirical: Cross-sectional survey	HIV and other communicable diseases	CPR, particularly MTM ventilation	Basic cardiac life-support instructors	United States
**Locke, Berg, Sanders, et al (1995)** ^20^					
Identify attitudes toward and potential obstacles to bystander CPR	Empirical: Cross-sectional survey	HIV and other communicable diseases	CPR	Clinicians and laypeople	United States
**Hew, Brenner, & Kaufman (1997)** ^21^					
Examination of the relationship between perceived risk and fear of contracting infectious diseases and the willingness of paramedics and EMTs to perform MTM ventilation	Empirical: Cross-sectional survey	HIV and other communicable diseases	CPR, particularly MTM ventilation	EMTs and paramedics	United States
**Bevan & Upshur (2003)** ^22^					
Perspective on the ethics of practicing anesthesia on SARS patients	Conceptual: Commentary	SARS	Tracheal intubation and other anesthesia procedures	Anesthesiologists, respiratory technologists, intensive care staff, and emergency room personnel	Canada
**Torabi-Parizi, Davey, Suffredini, & Chertow (2015)** ^23^					
Consideration of ethical and practical aspects of caring for patients critically ill with EVD	Conceptual: Commentary	Ebola Virus Disease	Critical care procedures (eg, CPR; endotracheal intubation; mechanical, noninvasive, and bag-mask ventilation)	Inpatient facility personnel	West Africa
**Yilmaz & Ozbilen (2020)** ^24^					
Assessment of knowledge, behaviors, and anxiety levels of orthodontists during the COVID-19 pandemic	Empirical: Cross-sectional survey	COVID-19	Orthodontic procedures including debonding	Orthodontists	Turkey
**Yu, Teh, & Aung (2020)** ^25^					
Raise awareness of the impact COVID-19 had on procedure-based specialty training	Conceptual: Commentary	COVID-19	Bronchoscopies, intubation, and other unspecified AGPs	Medical trainees	Australia
**Chong, Chen, Lien, et al (2021)** ^26^					
Evaluate the attitude and behavior toward bystander CPR during the COVID-19 pandemic	Empirical: Cross-sectional survey	COVID-19	CPR	Healthcare providers and laypeople	Taiwan
**Elliott, Ochieng, Jepson, et al (2021)** ^27^					
Present risk mitigation innovations developed by international surgical teams during the early stages of the COVID-19 pandemic	Empirical: Semi-structured interviews	COVID-19	Unspecified surgical AGPs	Surgeons, anesthesiologists, and nurses	United Kingdom, Italy, Spain, New Zealand, China, and the United States
**Johnson, Sullivan, Rastegar, & Gurubhagavatula (2021)** ^28^					
Assess sleep medicine practitioners’ concerns about COVID-19	Empirical: Cross-sectional survey	COVID-19	Aerosol-generating positive airway pressure devices (eg, CPAP)	Sleep medicine physicians and technologists	United States
**Marya, Karobari, Selvaraj, et al (2021)** ^29^					
Analyze dentists’ infection risk perception and their efforts to prevent the spread of COVID-19	Empirical: Cross-sectional survey	COVID-19	Unspecified dental AGPs	Dentists	Italy, India, Malaysia, Saudi Arabia, Cambodia, and Thailand
**Sari, Helmi, Kurniawaty, Wisudarti, Meliala, & Trisnantoro (2021)** ^30^					
Investigate doctors’ decision to perform CPR in COVID-19 patients under scarce resources	Empirical: Cross-sectional survey	COVID-19	CPR	ICU physicians	Indonesia
**Al-Shiakh, Tran, Caggiula, Berezowski, Barnawi, & Pourmand (2022)** ^31^					
Describe, characterize, and address attitudes and concerns of healthcare workers toward CPR of out-of-hospital cardiac arrests patients during the COVID-19 pandemic	Empirical: Cross-sectional survey	COVID-19	CPR	Attending and resident physicians, physician assistants, nurse practitioners, medical students, emergency medical technicians, registered nurses, and auxiliary medical staff	United States and Saudi Arabia
**Burstyn & Holt (2022)** ^32^					
Identify workplace factors that place physicians and nurses at risk for anxiety and depression during the COVID-19 pandemic	Empirical: Cross-sectional survey	COVID-19	Unspecified	Physicians and nurses	United States
**Cousins, Patel, Araujo, et al (2022)** ^33^					
Determine the provision of AGPs and capture dental practitioners’ beliefs and concerns	Empirical: Cross-sectional survey	COVID-19	Unspecified dental AGPs	Dentists, dental nurses, and dental hygienists/therapists	United Kingdom
**Varoni, Cinquanta, Rigoni, et al (2022)** ^34^					
Investigate the impact of the COVID-19 pandemic on dental hygienists’ attitudes, work routine, and socioeconomic profile	Empirical: Cross-sectional survey	COVID-19	Dental procedures including scaling, polishing, air-polishing, manual scaling and root planing, sealants, bleaching, laser therapy, and periodontal probing/charting	Dental hygienists	Italy

Note. AGPs, aerosol-generating procedures; CPAP, continuous positive airway pressure; CPR, cardiopulmonary resuscitation; EMT, emergency medical technician; EVD, Ebola virus disease; HIV, human immunodeficiency virus; ICU, intensive care unit; MTM, mouth-to-mouth; SARS, severe acute respiratory syndrome.

### Disease of interest

Across the 16 reports included in this review, 4 specific diseases were highlighted: (1) human immunodeficiency virus (HIV; *n* = 3), (2) severe acute respiratory syndrome (SARS; *n* = 1), (3) Ebola virus disease (EVD; *n* = 1), and (4) SARS-CoV-2 (COVID-19; *n* = 11). Though all reports tended to refer to a single illness as the primary disease of interest, several expected their discussion to apply to communicable respiratory diseases more generally.

The diseases highlighted in the reports mirrored the contemporary epidemics or public health crises at the times of publication. For example, studies conducted in the 1990s^19–21^ focused on HIV, whereas contemporary reports generally centered on COVID-19.^24–34^ Similarly, Bevan and Upshur^22^ published their SARS report in 2003 and the 2015 paper by Torabi-Parizi et al^23^ focused on EVD.

### Population of interest: Types of HCWs

Reports addressed different types of HCWs in various outpatient and inpatient settings. In total, 6 reports addressed clinicians and healthcare providers (including attending and resident physicians, nurse practitioners, registered nurses), medical technicians, auxiliary medical staff, basic cardiac life-support instructors, and emergency medical technicians and paramedics providing bystander cardiopulmonary resuscitation (CPR) in community settings or inpatient settings under conditions of scarce resources.^19–21,26,30,31^ Also, 3 reports focused on inpatient facility personnel such as anesthesiologists, surgeons, intensive care personnel, emergency room personnel, respiratory care personnel and nurses engaged in performing procedures such as endotracheal intubation, mechanical ventilation, general anesthesia, and renal replacement therapy.^22,23,27^ One additional report addressed medical trainees learning to perform these types of critical-care inpatient procedures such as endotracheal intubation and bronchoscopy.^25^ Furthermore, 4 reports addressed dentistry professionals including orthodontists, dentists, dental nurses, and dental hygienists and hygiene therapists.^24,29,33,34^ One report studied sleep medicine physicians and technologists,^28^ and another focused generally on physicians and nurses in inpatient settings.^32^

### AGPs of interest

The cumulative list of AGPs addressed across all 16 reports included CPR; anesthesia procedures including intubation (endotracheal, mechanical, noninvasive, and bag mask); bronchoscopies; orthodontic and dental procedures including bonding, scaling, polishing, root planning, bleaching, laser therapy, and periodontal probing and/or charting; and positive airway pressure.

Cardiopulmonary resuscitation (CPR, including mouth-to-mouth ventilation, which was recommended for use during CPR until 2008)^35^ was the single most commonly explored AGP in our review. All 6 of these reports were empirical cross-sectional survey investigations of HCWs attitudes and behaviors surrounding CPR during outbreaks of infectious disease.^19–21,26,30,31^

The remaining reports referred to 2 or more AGPs categorized by purpose or discipline. Of these, dental and orthodontic procedures were most commonly considered (*n* = 4).^24,29,32,34^ One study reported attitudes regarding the use of aerosol-generating positive airway pressure devices (eg, continuous positive airway pressure [CPAP]).^28^ Another report did not specify any particular AGP.^32^ The remaining 4 reports explored unspecified critical care, anesthesia, and surgical procedures.^22,23,25,27^

### Infection risk perceptions

Collectively, the included studies suggest that AGPs are perceived to place HCWs at high risk of becoming infected with respiratory pathogens (Table 2). Conceptual evidence suggests that anesthesiologists,^22^ critical care providers,^23^ and medical trainees^25^ are at increased risk for infection with respiratory illnesses when conducting AGPs. Empirical work suggests that most sleep medicine practitioners were extremely or very concerned about transmission of an infectious disease during sleep studies.^28^ Furthermore, paramedics and emergency medical technicians (EMTs),^21^ basic life-support instructors,^19^ HCWs,^20,31^ and laypeople^20,26^ all perceive an elevated infection risk when conducting CPR. Evidence from these studies also suggests that risk perception influences HCW attitudes toward and emotional response to conducting CPR during an outbreak.^19–21,26,30^

**Table 2.** Findings from Selected Peer-Reviewed Publications

Reference	Infection Risk Perception	Affective Response
**Empirical publications**		
Ornato, Hallagan, McMahan, Peeples, & Rostafinski (1990)^19^	More than 50% of BCLS instructors believed there was at least some risk of acquiring AIDS from ventilating a manikin. Almost all BCLS instructors believed that working in an emergency department or on an ambulance imposed a significant risk. More than 50% of BCLS instructors believed that ventilating a CPR manikin was a hazard (generally a small-to-medium risk).	Fear of being exposed to a disease would cause BCLS instructors to hesitate giving MTM ventilation. Many BCLS instructors will not perform MTM ventilation on a stranger without a protective barrier device. Most instructors would not perform or might hesitate to perform MTM ventilation on “high-risk” victims such as a heroin drug-overdose victim (90%), a man on a San Francisco bus (82%), or at a New York City football game (71%), or a young hemophiliac (65%).
Locke, Berg, Sanders, et al (1995)^20^	Clinicians and laypeople perceive a risk of infection from CPR, particularly MTM ventilation.	82% of respondents were “very concerned” or “moderately concerned” about the possibility of disease transmission during MTM ventilation. Women were significantly more concerned than men. Laypeople were significantly more concerned than clinicians. Concerns about disease transmission was negatively correlated with willingness to perform MTM ventilation in both strangers and close friends and relatives. It was not correlated with willingness to perform chest compressions only. Only 15% of respondents would definitely perform CPR even though they were trained and no one else was available.
Hew, Brenner, & Kaufman (1997)^21^	EMTs and paramedics perceive a risk of infection from CPR, particularly MTM ventilation.	Fear of infection, fear of litigation, fear of vomitus, and finding performing CPR without a mask to be distasteful created unwillingness in EMTs and paramedics to perform MTM ventilation Most EMTs (57%–75%) and paramedics (96%) would not perform MTM ventilation on trauma victims, elderly individuals, suspected homosexuals, or whose HIV status was unknown. Some EMTs (37%) and paramedics (23%) would not perform MTM resuscitation on a child.
Yilmaz & Ozbilen (2020)^24^	87% of orthodontists thought they were at high risk for contracting COVID-19 at work.	16.7% of respondents demonstrated generalized anxiety disorder during the COVID-19 pandemic, though this cannot be directly linked to performance of AGPs. Most participants avoided debonding procedures (97%), bonding broken attachments (92%), using high-speed handpieces with water (97%) or without water (90%), using low-speed handpieces (89%), and 3-way syringe (93%).
Chong, Chen, Lien, et al (2021)^26^	None reported.	61% (*n* = 822) of all respondents said an emerging infectious disease outbreak had a negative impact on their attitude toward performing bystander CPR. Only 40.7% of HCPs had a negative change in attitude as compared to 74.4% of physicians and 70.9% of laypersons. Attitudes towards bystander CPR influenced the likelihood respondents would participate in CPR: • 5.1% of HCPs, 14.4% of physicians, and 11.6% of laypersons would absolutely refuse to provide CPR. • 0.2% of HCPs, 0% of physicians, and 16.4% of laypersons would perform CPR with instruction. • 32.0% of HCPs, 33.3% of physicians, and 17.7% of laypersons would perform CPR with a face mask • 35.8% of HCPs, 48.9% of physicians, and 41.5% of laypersons would perform CPR without MTM • 26.9% of HCPS, 3.3% of physicians, and 12.8% of laypersons would perform CPR under any circumstance
Elliott, Ochieng, Jepson, et al (2021)^27^	The perception of risks during a pandemic such as COVID-19 can be complex and context dependent. Participants described how new national and local guidelines had state that AGPs, such as laparoscopic and endoscopic approaches, subjected staff to elevated risk. However, many reflected there had been some uncertainty as to what constitutes an AGP. There were conflicting views as to whether alternative techniques should be adopted.	New, uncertain, and unfamiliar risks paired with uncertainty as to precisely what constituted an AGP has significant impact on HCPs including experiences of fear and anxiety about personal safety, psychological burden, and risk of burnout.
Johnson, Sullivan, Rastegar, & Gurubhagavatula (2021)^28^	Most respondents were extremely or very concerned about sleep study practices: • 82.7% of respondents concerned about droplet transmission from positive airway pressure devices. • 81.7% of respondents were concerned about exposing sleep technicians to COVID-19. • 81.7% of respondents were concerned about airborne transmission from positive airway pressure devices. • 72.6% of respondents were concerned about contaminated surfaces. • 70.8% of respondents were concerned about exposing patients to COVID-19. • 63.5% of respondents were concerned about shared room ventilations systems. • 61.5% of respondents were concerned about droplet transmission from oxygen.	Greater than 40% of both sleep physicians and technologists felt aerosol precautions in the form of PPE were always important for laboratory studies in patients with unknown COVID-19 viral status. Also, 20%–30% of sleep physicians and technologists felt that aerosol precautions in the form of PPE were important for laboratory studies only with positive airway pressure devices.
Marya, Karobari, Selvaraj, et al (2021)^29^	39.1% of dentists were apprehensive about working under stressful conditions and perceived the situation as high risk. 46% of dentists felt they had become accustomed to the situation and it was acceptable to work as they saw the current scenario as a low-risk situation. 14.9% of dentists felt it was no risk.	None reported.
Sari, Helmi, Kurniawaty, Wisudarti, Meliala, & Trisnantoro (2021)^30^	No data reported. Presumed that CPR is risky as a premise for study.	Approximately 85% of ICU physicians reported experiencing moderate to extreme stress when performing CPR under scarce resources. The majority (61%) of ICU doctors decided not to perform CPR in situations of scarce resources.
Al-Shiakh, Tran, Caggiula, Berezowski, Barnawi, & Pourmand (2022)^31^	2% (*n* = 10 of 501) of participants believed that CPR carries minimal-to-low risk of COVID-19 transmission. 25% (*n* = 126 of 501) of participants were concerned about contracting COVID-19 during CPR and 16% were concerned about transmitting COVID-19 to others during CPR. Participants reported one or multiple transmission pathways for COVID-19 based on their baseline medical knowledge: • Droplet (96%, *n* = 481) • Close contact with an infected person (65%, *n* = 325) • Touching contaminated surfaces (49%, *n* = 243) • Airborne (44%, *n* = 218)	Fear played an important role in unwillingness to conduct CPR. 66% of participants (*n* = 331 of 501) were willing to perform bystander CPR during the pandemic. Willingness to perform CPR included the following reasons: • Medical oath or moral responsibility (94%, *n* = 311 of 331) • Belief that CPR is minimal-to-low risk (3%, *n* = 10 of 331) • Legal liability (1%, *n* = 3 of 331) 34% (*n* = 170 of 501) were unwilling (12%, *n* = 58 of 501) or uncertain about their willingness (22%, *n* = 111) to perform bystander CPR. This group’s reasons for their unwillingness or uncertainty included: • Fear of contracting COVID-19 (74%, *n* = 126 of 170) • Fear of transmitting COVID-19 to others (46%, *n* = 78 of 170) • PPE unavailability (25%, *n* = 43 of 170) • Belief that out of hospital cardiac arrest from COVID-19 has a low survival rate (21%, *n* = 36 of 170) • Absence of effective treatment or vaccines (16%, *n* = 27 of 170) • Fear of isolation and not being able to work for 14 days (12%, *n* = 20 of 170) Participants believed the following precautions would improve confidence in performing bystander CPR: • Including PPE with AEDs in public places (88%, *n* = 442) • Mandatory COVID-19 test for bystanders performing CPR (41%, *n* = 204) • Mandatory isolation for bystanders when testing is unavailable (21%, *n* = 107)
Burstyn & Holt (2022)^32^	None reported.	There was no evidence that participation in AGPs was associated with depression or anxiety in physicians. Participation in AGPs was associated with increased cases of anxiety in nurses at 1 of the 2 hospitals sampled. There was no evidence that participation in AGPs was associated with increased depression in nurses.
Cousins, Patel, Araujo, et al (2022)^33^	Dentists expressed doubts about the severity of risk, citing several reasons: • Risk of transmission of COVID-19 in the dental setting was described by some respondents as minimal due to screening processes and pre-existing cross-infection control measures. • The impression that dental AGPs are not the same risk level as AGPs in other professions: ‘I do think comparing dental aerosol (which is vast majority from dental lines and in our case is antimicrobial) with aerosol from intubation (100% from patient) is ludicrous!’(dentist). • Infectious diseases such as Hepatitis B, Hepatitis C, HIV, SARS, MERS, and Influenzas had not affected dental practices disproportionately nor were precautionary measures taken. If aerosol transmission of COVID-19 was an issue, they would expect to have seen clusters of infection in dental practices during the months of January to March 2020, before extra preventative measures were taken.	Attitudes and emotional responses to AGPs were not reported. Emotional responses detailed related to the pandemic in general. Respondents highlighted the toll that the pandemic was taking both personally and professionally on dental professionals: • Frustration over what was felt to be over-regulation of the profession and prevention of dental professionals being supported to exercise their clinical judgement. • Concerns around staff health and safety and the challenges of working under the current levels of PPE: (1) ‘It is very stressful to work in the full PPE and adds considerable stress to an already stressful job’(dentist), (2) ‘Undertaking AGPs with the current level of PPE is extremely challenging for the staff and may theoretically reduce risk of transmission but poses other threats in terms of health and wellbeing of the staff.’ (dentist) • Emotional wellbeing of dental professionals was perceived to be negatively impacted, ‘the mental health of dentists has suffered terribly.’ (dentist) • Concern surrounding the impact of reduced practice capacity on patient care. Anxiety that patients came to harm because of not being able to carry out routine fillings or screening for malignant diseases.
Varoni, Cinquanta, Rigoni, et al (2022)^34^	Dental procedures perceived as high risk for spreading infection included: (1) air polishing (98%, *n* = 307), (2) scaling with sonic/ultrasonic instruments (85%, *n* = 266), (3) polishing (37%, *n* = 115), (4) sealants (3%, *n* = 9), (5) dental bleaching (2%, *n* = 6), (6) laser-therapy (2%, *n* = 6), (7) manual scaling and root planing (2%, *n* = 5), (8) air or water syringe use (0.3%, *n* = 1).	Most participants (75%, *n* = 234) were afraid of becoming infected with COVID-19 during clinical practice. Approximately half (58%, *n* = 180) were afraid of treating patients having symptoms attributable to COVID-19. A fifth (21%, *n* = 67) of participants thought about changing their job.
**Conceptual publications**		
Bevan & Upshur (2003)^22^	In the absence of specific treatment or vaccination, anesthesiologists are at increased risk of contracting SARS, particularly during tracheal intubation. Precautions that can prevent spread must be identified and implemented. Donning PPE creates an unacceptable response time during acute cardiac arrest. A ‘Protected Code Blue’ protocol may enable rapid tracheal intubation of patients with highly infectious diseases.	Anesthesiologists risk extreme moral distress. They fear infection, passing SARS to family, and the economic consequences of illness. Anesthesiologists’ moral values often dictate that they should treat SARS patients without regard to their own safety. Widely adopted effective tracheal intubation protocols should reduce fear.
Torabi-Parizi, Davey, Suffredini, & Chertow (2015)^23^	Provision of CPR to critically ill patients with EVD poses an unacceptably high risk to HCWs. Procedures including blood draws, venous or arterial catheter placement, or tissue manipulation, such as endotracheal intubation, carry the risk of PPE barrier breach and possible skin or mucosal surface exposure. The potential for transmission from infectious upper respiratory tract secretions is of significant concern. AGPs, including endotracheal intubation, noninvasive ventilation, and bag-mask ventilation prior to intubation, were associated with increased transmission risk.	Social stigma, fear, and knowledge gaps in disease pathogenesis and transmission risk have contributed to some providers electing against participation in the care of patients with EVD or establishing a priori limits in patient-care interventions.
Yu, Teh, & Aung (2020)^25^	AGPs, such as bronchoscopies, are considered high-risk for infection spread. Now performed with enhanced personal protective equipment. General anesthesia and close-circuit ventilation are currently recommended for most airway procedures.	None reported.

Note. AGPs, aerosol-generating procedures; AIDS, acquired immunodeficiency syndrome; BCLS, basic cardiac life support; CPR, cardiopulmonary resuscitation; EMT, emergency medical technician; EVD, Ebola virus disease; HCP, healthcare provider; HCW, healthcare worker; MTM, mouth-to-mouth; SARS, severe acute respiratory syndrome.

Dentistry practitioners appear to have mixed views regarding the risk of transmitting an infectious disease during their practice. One study showed that 87% of orthodontists believe that they are at high-risk for contracting COVID-19 while conducting AGPs such as bonding and debonding procedures.^24^ Similarly, in another study, most dentists perceived air polishing and scaling with sonic and ultrasonic instruments to be high-risk AGPs related to their practice.^34^ Despite this finding, another study showed that 39% of dentists perceived their jobs to be high risk, 46% viewed them as low risk, and 15% felt there was no risk.^29^ Dentists cited several reasons for doubting risk severity, including the perception that dental AGPs are not the same risk level as AGPs in other professions and that the screening processes and pre-existing cross-infection control measures sufficiently protected against transmission.^33^

### Affective response to perceptions of infection risk

Common affective responses to infection risk perceptions include fear of infection,^19–22,27,31,34^ fear of economic consequences of illness,^22^ fear of litigation,^21,31^ fear of spreading the disease to others,^21,31^ and unwillingness to perform AGPs.^19–21,23,24,30,31^ Performance of AGPs during an infectious outbreak can create emotional stress responses that impact the psychological well-being of HCWs^27^ and influence the decision to perform potentially lifesaving AGPs.^19,21,24,26,30^

## Discussion

To our knowledge, this is the first review of HCW perceptions of AGP risk and affective response to conducting AGPs, including during outbreaks of high-consequence pathogens. Key takeaways of the included reports indicate that HCWs perceive increased risk to contracting a high-consequence infectious disease from participating in an AGP and that this perception stimulates a negative affective response and hesitancy to participate in the procedures. As Bevan and Upshur wrote, “Communicable diseases underscore human vulnerability and a fearful response to contagion is understandable, particularly in a context where the infectious agent is poorly understood, and the science is evolving.”^22^

However, our findings indicate a paucity of data-driven research on AGP risk perceptions and affective response. Our review demonstrates that HCWs perceive AGPs as an occupational hazard that may pose substantial personal and vocational consequences. Still, very little research has been conducted to quantify the degree of perceived risk (ie, how risky various AGPs are perceived to be). Furthermore, precision of the science is weak: numerous AGPs have been implicated as risky (eg, intubation or extubation, CPR, orthodontic bonding); however, few studies have characterized exposure and transmission risk during AGPs. The lack of sufficient empiric data to adequately characterize exposure or transmission risk of high-consequence pathogens during AGPs, or even which procedures should be considered AGPs because they generate potentially infective small particle aerosols from patients, leads to uncertainty among HCWs. This uncertainty causes anxiety, fear, and worry, potentially decreasing workforce wellness and workplace satisfaction^27^ which, in turn, may contribute to workforce attrition.^4,36,37^ Faced with uncertainty, many HCWs may assume an elevated risk of infection (including secondary transmission to family, friends, and coworkers), which creates a conflict between duty to care versus self-protection.

When clinical evidence informing self-protection is lacking, HCWs will likely search for ways to limit infection risk. Protection motivation theory proposes that people will protect themselves based on appraisals of threat (ie, situation severity) and coping (ie, response option).^38,39^ Threat appraisal involves an assessment of both the likelihood a situation will emerge and the severity of the situation if it occurs. Coping appraisal involves assessments that a particular behavior will reduce the threat (‘response efficacy’) and the belief that one can carry out an effective response (‘self-efficacy’). Our review underscores the concept that AGPs are generally believed to incur high infection risk. How HCWs choose to cope with this appraisal depends on what they perceive to be effective and acceptable response options and whether they believe they can and are willing to commit to those actions.

For example, HCWs rely on personal protective equipment (PPE) to prevent the spread of infection.^25,28^ However, PPE alone is an insufficient solution. First, PPE use is rife with potential failure modes such as improperly fitted or functioning PPE and human error.^40^ Thus, the effectiveness of PPE is highly dependent on the institutional framework for supporting proper PPE use through user training, fit testing, and provision of ample supplies. For these reasons, it is the lowest-ranked intervention to reduce hazardous exposures in the hierarchy of controls.^41^ Second, donning and doffing procedures can be time intensive, creating unacceptable response lags to emergencies.^22^ Better infection controls, such as engineering controls to capture and remove infectious aerosols at the source and validated knowledge of infection risk, will facilitate the use of PPE only when necessary, reducing resource waste and time lags. Other response options, such as social distancing and reducing or eliminating certain AGPs, may not be seen as acceptable from the standpoint of patient welfare or safety, even if they are deemed effective for slowing the spread of infections. Practicing clinicians place a premium on meeting accepted standards of care. Distress may result, particularly when HCWs perceive a lack of agency and control.

Self-determination theory suggests that autonomy is 1 of 3 essential human needs underlying intrinsic motivation.^42^ Developing a sense of autonomy and control over situations is fundamental for individuals to be able to self-regulate or maintain and internalize recommended behaviors such as respecting rules, complying with legal requirements, or adhering to medical treatment plans.^43^ Perception of control plays a crucial role in how people formulate judgments and make decisions about risk. Nordgren et al^42^ differentiated between risk control (ie, command over the result) and volition (ie, command over risk exposure). Although people tend to underestimate risk outcomes which they perceive to control,^44–46^ volition increases risk perceptions.^44^ When an individual can influence their risk exposure, the risk is potentially avoidable and, therefore, voluntary. Control, on the other hand, reflects an ability to prevent negative outcomes (eg, infection) once risky behavior has been initiated. Although many people generally find voluntary risks more acceptable,^47^ regret may arise in the event of negative outcomes.^44^ People tend to avoid or delay decisions they may regret, which they can anticipate and account for in the decision-making process. Yet, prompt decision making and early intervention can improve clinical outcomes.^48,49^

Nuanced understanding of how these phenomena interrelate and unfold is critical in our mission to create safer healthcare environments for both patients and workers. As the COVID-19 pandemic has highlighted, risk perceptions powerfully influence behavioral choices.^50^ The results of our review point to the need for more empirical work to thoroughly understand the interplay between HCW risk perceptions of distinct AGPs, affective responses to conducting these procedures under various conditions, and the resulting decision to participate in these procedures. Studying the intricacies of HCW perceptions regarding the volition and control they experience while conducting specific AGPs as well as the severity of the risk they believe various AGPs pose, is essential for advancing clinical practice. Results from such studies will point to methods for mitigating provider distress and better recommendations for when and how to conduct AGPs.

Our review had several limitations. The small sample size limits the power and generalizability of our findings. AGPs and pathogens of focus as well as geographical location varied significantly among the included studies. These variances, especially within such a small sample, can cloud our understanding of perceived risks and the resulting emotional response. The various pathogen types represented in our sample are not all respiratory diseases (eg, HIV), and their perceived risk during performance of AGPs, may be significantly lower than for respiratory diseases. These limitations in our results underscore the importance of rigorously designed and conducted clinical studies that distinguish and quantify occupational risks posed by AGPs, including a clear understanding of procedures that do, and do not, produce aerosols or otherwise increase the risk of airborne transmission. Natural experiments and observational studies with ambiguous clinical significance are hypothesis generating but do not sufficiently answer essential clinical questions about person-to-person transmission to resolve clinicians’ uncertainties about the risks of AGPs. Sufficiently powered, robust, prospective studies with clinical end points designed to achieve unequivocal answers for specific AGPs, pathogens, and the context in which AGPs are performed (emergent vs elective) are an important resource for those who perform AGPs.

In conclusion, the perception of AGP risk is complex and context dependent. New and unfamiliar hazards paired with uncertainty regarding what precisely constitutes an AGP and how to appropriately mitigate risk has a significant effect on HCWs including experiences of fear and anxiety about personal and others’ safety.^27^ These fears may create a psychological burden conducive to burnout. More research is needed to appropriately categorize medical procedures as AGPs, to document the actual risks to HCWs performing or in proximity to the performance of AGPs, to empower HCWs to decide when and how to safely perform these procedures, and to support HCWs experiencing a negative emotional reaction to the psychological burden of their decisions.

## References

[1] Gilleen J , Santaolalla A , Valdearenas L , Salice C , Fusté M. Impact of the COVID-19 pandemic on the mental health and well-being of UK health workers. BJPsych Open 2021;7:e88. 33910674 10.1192/bjo.2021.42 PMC8082128

[2] Ibar C , Fortuna F , Gonzalez D , et al. Evaluation of stress, burnout and hair cortisol levels in health workers at a university hospital during COVID-19 pandemic. Pychoneuroendocrinology 2021;128:105213. 10.1016/j.psyneuen.2021.105213 PMC8015376 33845387

[3] Shreffler J , Petrey J , Huecker M. The impact of COVID-19 on healthcare worker wellness: a scoping review. West J Emerg Med 2020;21:1059–1066. 32970555 10.5811/westjem.2020.7.48684 PMC7514392

[4] Mehta S , Machado F , Kwizera A , et al. COVID 19: a heavy toll on healthcare workers. Lancet Respir Med 2021;9:226–228. 33556317 10.1016/S2213-2600(21)00068-0 PMC7906726

[5] Cox JW , Miller ME , Jamison P. ‘Dad, are you okay?’: doctors and nurses fighting pandemic fear infecting their families. The Washington Post website. https://www.washingtonpost.com/local/dad-are-you-okay-doctors-and-nurses-fighting-pandemic-fear-infecting-their-families/2020/03/18/8beefc66-689b-11ea-b313-df458622c2cc_story.html. Published March 18, 2020. Accessed June 23, 2022.

[6] Cawcutt KA , Starlin R , Rupp ME. Fighting fear in healthcare workers during the COVID-19 pandemic. Infect Control Hosp Epidemiol 2020;41:1192–1193. 32580790 10.1017/ice.2020.315 PMC7338429

[7] Transmission of SARS-COV-2: implications for infection prevention precautions. World Health Organization website. https://www.who.int/news-room/commentaries/detail/transmission-of-sars-cov-2-implications-for-infection-prevention-precautions. Published July 9, 2020. Accessed June 23, 2022.

[8] Joint consensus statement on addressing the aerosol transmission of SARS CoV-2 and recommendations for preventing occupational exposures: fact sheet. American Industrial Hygiene Association website. https://aiha-assets.sfo2.digitaloceanspaces.com/AIHA/resources/Fact-Sheets/Joint-Consensus-Statement-on-Addressing-the-Aerosol-Transmission-of-SARS-CoV-2-Fact-Sheet.pdf. Published 2020. Accessed June 23, 2022.

[9] Clinical questions about COVID-19: questions and answers. Centers for Disease Control and Prevention website. https://www.cdc.gov/coronavirus/2019-ncov/hcp/faq.html?CDC_AA_refVal=https%3A%2F%2Fwww.cdc.gov%2Fcoronavirus%2F2019-ncov%2Fhcp%2Finfection-control-faq.html. Published November 17, 2021. Accessed June 23, 2022.

[10] National infection prevention and control manual for England. Published June 9, 2022. National Health Services England website. https://www.england.nhs.uk/wp-content/uploads/2022/04/C1636-national-ipc-manual-for-england-v2.pdf. Accessed June 23, 2022.

[11] Tran K , Cimon K , Severn M , Pessoa-Silva CL , Conly J. Aerosol-generating procedures and risk transmission of acute respiratory infections to healthcare workers: a systematic review. PLoS One 2012;7:e35797. 22563403 10.1371/journal.pone.0035797 PMC3338532

[12] Wilson NM , Marks GB , Eckhardt A , et al. The effect of respiratory activity, non-invasive respiratory support and face masks on aerosol generation and its relevance to COVID-19. Anaesthesia 2021;76:1465–1474. 33784793 10.1111/anae.15475 PMC8250912

[13] Klompas M , Milton DK , Rhee C , Baker MA , Leekha S. Current insights into respiratory virus transmission and potential implications for infection control programs: a narrative review. Ann Intern Med 2021;174:1710–1718. 34748374 10.7326/M21-2780

[14] Harding H , Broom A , Broom J. Aerosol-generating procedures and infective risk to healthcare workers from SARS-CoV-2: the limits of the evidence. J Hosp Infect 2020;105:717–725. 32497651 10.1016/j.jhin.2020.05.037 PMC7263217

[15] Savage LJ. The Foundations of Statistics. New York, NY: Wiley; 1954.

[16] Attema AE , Bleichrodt H , L’Haridon O. Ambiguity preferences for health. Health Econ 2018;27:1699–1716. 29971896 10.1002/hec.3795 PMC6221042

[17] Dohmen T , Falk A , Huffman D , Sunde U , Schupp J , Wagner GG. Individual risk attitudes: measurement, determinants, and behavioral consequences. J Eur Econ 2011;9:522–550.

[18] Reibstein DJ , Lovelock CH , Dobson RDP. The direction of causality between perceptions, affect, and behavior: an application to travel behavior. J Consum Res 1980;6:370–376.

[19] Ornato JP , Hallagan LF , McMahan SB , Peeples EH , Rostafinski AG. Attitudes of BCLS instructors about mouth-to-mouth resuscitation during the AIDS epidemic. Ann Emerg Med 1990;19:151–156. 2301792 10.1016/s0196-0644(05)81800-1

[20] Locke CJ , Berg RA , Sanders AB , et al. Bystander cardiopulmonary resuscitation: concerns about mouth-to-mouth contact. Arch Intern Med 1995;155:938–943. 7726702 10.1001/archinte.155.9.938

[21] Hew P , Brenner B , Kaufman J. Reluctance of paramedics and emergency medical technicians to perform mouth-to-mouth resuscitation. J Emerge Med 1997;15:279–284. 10.1016/s0736-4679(97)00006-1 9258774

[22] Bevan JC , Upshur REG. Anesthesia , ethics, and severe acute respiratory syndrome. Can J Anesth 2003;50:977–982. 14656773 10.1007/BF03018359 PMC7090539

[23] Torabi-Parizi P , Davey RT Jr , Suffredini AF , Chertow DS. Ethical and practical considerations in providing critical care to patients with Ebola virus disease. Chest 2015;147:1460–1466. 25764372 10.1378/chest.15-0278 PMC4451704

[24] Yilmaz HN , Ozbilen EO. The assessment of knowledge, ehaviors, and anxiety levels of the orthodontists about COVID-19 pandemic. Turk J Orthod 2020;33:224–231. 33447465 10.5152/TurkJOrthod.2020.20128 PMC7771292

[25] Yu C , Teh BM , Aung AK. COVID-19 significantly affects specialty training. Int Med J 2020;50:1160–1161. 10.1111/imj.14975 PMC7461477 32827335

[26] Chong KM , Chen JW , Lien WC , et al. Attitude and behavior toward bystander cardiopulmonary resuscitation during COIVD-19 outbreak. PLoS One 2021;16:e0252841. 34161378 10.1371/journal.pone.0252841 PMC8221461

[27] Elliott D , Ochieng C , Jepson M , et al. ‘Overnight, things changed. Suddenly, we were in it’: a qualitative study exploring how surgical teams mitigated risks of COVID-19. BMJ Open 2021;11:e046662. 10.1136/bmjopen-2020-046662 PMC8210660 34135048

[28] Johnson KG , Sullivan SS , Rastegar V , Gurubhagavatula I. Sleep medicine healthcare-worker concerns about COIVD-19: an early pandemic survey. Respir Care 2021;66:1729–1738. 34433676 10.4187/respcare.09106

[29] Marya A , Karobari MI , Selvaraj S , et al. Risk perception of SARS-CoV-2 infection and implementation of various protective measures by dentists across various countries. Int J Environ Res Public Health 2021;18:5848. 34072456 10.3390/ijerph18115848 PMC8199051

[30] Sari D , Helmi M , Kurniawaty J , Wisudarti CFR , Meliala A , Trisnantoro L. Perceptions of ICU doctors in Indonesia regarding cardiopulmonary resuscitation for criticall ill patients with COVID-19. Int Med J 2021;28:485–486.

[31] Al-Shiakh S , Tran QK , Caggiula A , Berezowski I , Barnawi B , Pourmand A. Attitudes among healthcare professionals towards cardiopulmonary resuscitation during COVID-19. Am J Emerg Med 2022;52:34–42. 34861518 10.1016/j.ajem.2021.11.017 PMC8591860

[32] Burstyn I , Holt K. A cross-sectional survey of the workplace factors contributing to symptoms of anxiety and depression among nurses and phsyicians during the first wave of COVID-19 pandemic in two US healthcare systems. Ann Work Expo Health 2022;66:312–333. 34590682 10.1093/annweh/wxab085 PMC8500032

[33] Cousins M , Patel K , Araujo M , et al. A qualitative analysis of dental professionals’ beliefs and concerns about providing aerosol-generating procedures early in the COVID-19 pandemic. BMJ Open 2022;8:2. 10.1038/s41405-022-00094-9 PMC8758920 35031596

[34] Varoni EM , Cinquanta L , Rigoni M , et al. The impact of COVID-19 on the dental hygienists: a cross-sectional study in the Lombardy first-wave outbreak. PLoS One 2022;17:e0262747. 35108297 10.1371/journal.pone.0262747 PMC8809622

[35] Our lifesaving history. American Heart Association website. https://cpr.heart.org/en/about-us/history-of-the-american-heart-association. Published 2022. Accessed March 23, 2022.

[36] Majeed M , Irshad M , Bartels J. The interactive effect of COVID-19 risk and hospital measures on turnover intentions of healthcare workers: a time-lagged study. Int J Environ Res Public Health 2021;18:10705. 34682450 10.3390/ijerph182010705 PMC8536040

[37] Yong E. Why healthcare workers are quitting in droves. The Atlantic website. https://www.theatlantic.com/health/archive/2021/11/the-mass-exodus-of-americas-health-care-workers/620713/. Published November 16, 2021. Accessed March, 23 2022.

[38] Rogers RW. A protection motivation theory of fear appeals and attitude change. J Psychol 1975;91:93–114. 28136248 10.1080/00223980.1975.9915803

[39] Rogers RW. Cognitive and Physiological Processes in Fear Appeals and Attitude Change: A Revised Theory of Protection Motivation. New York, NY: Guilford; 1983.

[40] Gurses AP , Dietz AS , Nowakowski E , et al. Human factors-based risk analysis to improve the safety of doffing enhanced personal protective equipment. Infect Control Hosp Epidemiol 2018;40:178–186. 30520708 10.1017/ice.2018.292

[41] Hierarchy of controls. National Institute for Occupational Safety and Health website. https://www.cdc.gov/niosh/topics/hierarchy/default.html. Accessed 23 March 2022.

[42] Ryan RM , Deci EL. Self-determination theory and the facilitation of intrinsic motivation, social development, and well-being. Am Psychol 2000;55:68–78. 11392867 10.1037//0003-066x.55.1.68

[43] Ryan RM , Patrick H , Deci EL , Williams GC. Facilitating health behaviour change and its maintenance: interventions based on self-determination theory. Eur Health Psychol 2008;10:2–5.

[44] Nordgren LF , Van Der Pligt J , Van Harreveld, F. Unpacking perceived control in risk perception: the mediating role of anticipated regret. J Behav Decis Mak 2007;20:533–544.

[45] Thompson SC , Armstrong W , Thomas C. Illusions of control, underestimations, and accuracy: a control heuristic explanation. Psychol Bull 1998;123:143–161. 9522682 10.1037/0033-2909.123.2.143

[46] Beisswingert BM , Zhang K , Goetz T , Fang P , Fischbacher U. The effects of subjective loss of control on risk-taking behavior: the mediating role of anger. Front Psychol 2015;6:774. 26217244 10.3389/fpsyg.2015.00774 PMC4493906

[47] Starr C. Social benefit versus technological risk. Science 1969;165:1232. 5803536 10.1126/science.165.3899.1232

[48] Gupta S , Wang W , Hayek S , et al. Association between early treatment with tocilizumab and mortality among critically ill patients with COVID-19. JAMA Intern Med 2021;181:41–51. 33080002 10.1001/jamainternmed.2020.6252 PMC7577201

[49] Moscana A. Neuraminidase inhibitors for influenza. N Engl J Med 2005;353:1363–1373. 16192481 10.1056/NEJMra050740

[50] Bruine de Bruin W , Bennett D. Relationships between initial COVID-19 risk perceptions and protective health behaviors: a national survey. Am J Prev Med 2020;59:157–167. 32576418 10.1016/j.amepre.2020.05.001 PMC7242956

